# Towards the Industrial Production of Omega-3 Long Chain Polyunsaturated Fatty Acids from a Genetically Modified Diatom *Phaeodactylum tricornutum*

**DOI:** 10.1371/journal.pone.0144054

**Published:** 2015-12-14

**Authors:** Mary L. Hamilton, Joanna Warwick, Anya Terry, Michael J. Allen, Johnathan A. Napier, Olga Sayanova

**Affiliations:** 1 Department of Biological Chemistry and Crop Protection, Rothamsted Research, Harpenden, AL5 2JQ, United Kingdom; 2 Plymouth Marine Laboratory, Prospect Place, The Hoe, Plymouth, PL1 3DH, United Kingdom; Stazione Zoologica Anton Dohrn, Naples, ITALY

## Abstract

The marine diatom *Phaeodactylum tricornutum* can accumulate up to 30% of the omega-3 long chain polyunsaturated fatty acid (LC-PUFA) eicosapentaenoic acid (EPA) and, as such, is considered a good source for the industrial production of EPA. However, *P*. *tricornutum* does not naturally accumulate significant levels of the more valuable omega-3 LC-PUFA docosahexaenoic acid (DHA). Previously, we have engineered *P*. *tricornutum* to accumulate elevated levels of DHA and docosapentaenoic acid (DPA) by overexpressing heterologous genes encoding enzyme activities of the LC-PUFA biosynthetic pathway. Here, the transgenic strain Pt_Elo5 has been investigated for the scalable production of EPA and DHA. Studies have been performed at the laboratory scale on the cultures growing in up to 1 L flasks a 3.5 L bubble column, a 550 L closed photobioreactor and a 1250 L raceway pond with artificial illumination. Detailed studies were carried out on the effect of different media, carbon sources and illumination on omega-3 LC-PUFAs production by transgenic strain Pt_Elo5 and wild type *P*. *tricornutum* grown in 3.5 L bubble columns. The highest content of DHA (7.5% of total fatty acids, TFA) in transgenic strain was achieved in cultures grown in seawater salts, Instant Ocean (IO), supplemented with F/2 nutrients (F2N) under continuous light. After identifying the optimal conditions for omega-3 LC-PUFA accumulation in the small-scale experiments we compared EPA and DHA levels of the transgenic strain grown in a larger fence-style tubular photobioreactor and a raceway pond. We observed a significant production of DHA over EPA, generating an EPA/DPA/DHA profile of 8.7%/4.5%/12.3% of TFA in cells grown in a photobioreactor, equivalent to 6.4 μg/mg dry weight DHA in a mid-exponentially growing algal culture. Omega-3 LC-PUFAs production in a raceway pond at ambient temperature but supplemented with artificial illumination (110 μmol photons m^-2^s^-1^ **)** on a 16:8h light:dark cycle, in natural seawater and F/2 nutrients was 24.8% EPA and 10.3% DHA. Transgenic strain grown in RP produced the highest levels of EPA (12.8%) incorporated in neutral lipids. However, the highest partitioning of DHA in neutral lipids was observed in cultures grown in PBR (7.1%). Our results clearly demonstrate the potential for the development of the transgenic Pt_Elo5 as a platform for the commercial production of EPA and DHA.

## Introduction

Omega-3 long chain polyunsaturated fatty acids (LC-PUFAs) with 20 carbons or more in length containing three or more *cis-* double bond, particularly EPA and DHA, play essential roles in human nutrition, including during neonatal development and also in adult cardiovascular health. LC-PUFAs can be classified into two main families, omega-6 (or *n*-6) and omega-3 (or *n*-3) families, depending on the position of the first double bond proximal to the methyl end of the fatty acid. These two families are not inter-convertible and their metabolites have opposing physiological roles (For Refs see [1]). At present, the ratio of omega-6/omega-3 fatty acids in typical Western diet is about 25:1, thus indicating a current deficiency in omega-3 fatty acids.

Currently, the main dietary source of EPA and DHA is marine fish. Depletion of wild fish stocks, pollution of the marine environment and expansion of the aquaculture industry are all factors which dictate that new sustainable sources of EPA and DHA need to be developed. Marine algae are the primary producers of LC-PUFAs and represent a logical and promising alternative source to fish oils. However, the efficient production of high value products such as EPA and DHA from algae is expensive and significant efforts in strain development and cultivation technologies are still required to reduce the currently high production costs associated with algal biomass.

The demand to produce microbial DHA and EPA as an alternative to fish oil sources has stimulated significant research within the last few years [1, 2]. However, despite a large and diverse variety of microalgal species with the capability to produce EPA and/or DHA, only a few have demonstrated potential for industrial-scale application. One of the main reasons for this is insufficient culture productivity—low growth rates and cell densities ultimately resulting in poor product yields [3]. Microalga growth and concomitant fatty acid accumulation are affected by a variety of factors, such as temperature, nutrient, CO_2_ availability and light intensity.

A sustainable industrial LC-PUFAs production system requires an understanding of both microalgal physiology and lipid biochemistry for the successful cultivation and manipulation of suitable algal strains. Whilst a large variety of microalgal species produce EPA and DHA, only a few accumulate both omega-3 LC-PUFAs in the desired ratio and/or display growth properties amenable to industrial scale up. The marine diatom *P*. *tricornutum* has recently emerged as a potential source for the production of EPA [4–6]. It is a well-established organism in the aquaculture industry, has a rapid growth rate and accumulates triacylglycerol (TAG) up to 30% of its dry cell weight. Moreover, the *P*. *tricornutum* genome has been fully sequenced [7] and genetic tools are available for metabolic engineering [8, 9], making it particularly attractive for biotechnological applications. Previously, we have transgenically engineered the *P*. *tricornutum* strain to accumulate elevated levels of DHA by overexpressing heterologous genes encoding enzyme activities of the LC-PUFA biosynthetic pathway [10]. This was the first report of the metabolic engineering of the omega- trait in transgenic algae. This transgenic strain, referred to as Pt_Elo5, has been characterised only at laboratory scale where it retains the growth characteristics of the wild type strain, yet accumulates substantial levels of both EPA and DHA, making it particularly promising for the evaluation and incorporation into commercial omega-3 production pipelines. However, knowledge of the growth of genetically modified microalgae under controlled conditions at larger scales represents a significant technical barrier to commercialisation and is one of the factors that currently limit exploitation. Utilising bespoke contained growth facilities, we have assessed the omega-3 LC-PUFA production capability of Pt_Elo5, exploiting a pilot scale pharmaceutical-grade photobioreactor (550 litres), an open-pond style system (working volume 1250 litres), as well as smaller bubble column systems (3.5 litres) in order to identify and evaluate key factors amenable to optimisation. Representing a scale-up of more than four orders of magnitude over our initial laboratory studies, our trials aim to demonstrate the potential of this transgenic strain for industrial scale omega-3 LC-PUFAs production.

## Material and Methods

### Culture and growth conditions

*P*. *tricornutum* UTEX 646 and transgenic strain Pt_Elo5 [10] were used in all experiments. Four types of algal cultivation systems were used: 100 mL cultures in 250 mL flasks, 3.5 L bubble column (BC), a closed fence style photobioreactor (PBR) and a miniature raceway pond with artificial illumination. Small scale (100 mL) cultures were maintained as described previously (Hamilton et al., 2014) in F/2 medium under constant light at 65 μmol photons m^-2^ s^-1^ and agitated at 65 rpm. Bubble columns were constructed using 6063 anodised aluminium alloy; 110 mm clear polycarbonate tubing, with a working culture volume of 3.5 L. Light was supplied via 36 W Grolux fluorescent tube and 36 W 865 daylight fluorescent tube providing 80 μmol photons m^-2^s^-1^. Cultures were aerated by constant bubbling at 5 L min^-1^. The cultures were supplied with additional CO_2_ at a continuous flow rate of 0.05 L min^-1^. The fence style photobioreactor was constructed by Bouygues Energies and Services (Manchester, UK) and had a working volume of approximately 550 L. An array of horizontal polycarbonate 50 mm diameter tubes (36 tubes in total, in a 6 tube manifold formation) served as the photostage and was illuminated by eight 600 W high pressure sodium lamps providing 450 μmol photons m^-2^s^-1^. Stainless steel (316S) pipework to a holding tank allowed the continuous circulation, via a centrifugal pump (up to 30 m^3^h^-1^), of the algal culture. The holding tank (200 litre L working volume) was continually sparged with air to aid oxygen removal (~10 L min^-1^), while carbon dioxide delivery was controlled through a pH stat system. Culture monitoring was performed via a Profilux 3 interface and included oxygen saturation, pH, conductivity and temperature measurement. Raceway ponds (RP) were located indoors and comprised 1250 L working volume; rectangular (3 m × 1.5 m) fibreglass tanks (sloped bottom); culture max. depth: 0.3 m; culture min. depth: 0.20 m; single baffle (2 m length). Constant agitation was achieved using 16 peristaltic pumps (EHEIM powerhead 650) with a laminar liquid flow velocity c. 0.05 m s^-1^. In addition to ambient light, ponds were supplemented by halogen bulbs, LED strips and fluorescent tubes; light level up to 110 μmol photons m^-2^ s^-1^ and were housed under polythene sheeting for culture/environment protection.

Cultures grown in 3.5 L Bubble Columns (BC), were prepared from ESAW basal salts (ES) and ESAW nutrients (EN), ES supplemented with F/2 nutrients (F2N), from commercial seawater salts, Instant Ocean (IO), supplemented with either EN (IO+EN) or with F2N (IO+F2N).

Cultures grown in BC were started with an addition of either wild type (WT) *P*. *tricornutum* or transgenic Pt_Elo5 inoculums (5%) to give a starting cell concentration of c. 5 x 10^5^ cells mL^-1^. Cell counts were conducted daily and the biomass was harvested by centrifugation. Cultures were grown at 20°C in constant illumination (24 h) or in cycles of 16 h of light: 8 h dark photoperiods (16:8h).

Cultures in the fence style photobioreactor (PBR) were grown in IO supplemented with F/2 nutrients at 20°C in 16:8 h cycle. The PBR was inoculated with Pt_Elo5 strain (5%) to give a starting cell concentration of c. 5 x 10^5^ cells mL^-1^.

Cultures in the RP systems were grown in medium prepared from Natural Sea Water (NSW, collected from the Western Channel Observatory (WCO) coastal station ‘L4’ and UV sterilised prior to innoculation) supplemented with F/2 nutrients. The RP was inoculated with Pt_Elo5 strain (5%) to give a starting cell concentration c. 5 x 10^5^ cells mL^-1^.

Analysis of the wild-type and transgenic algae have been performed during stationary growth phases. Growth stage was determined by triplicate cell counts using a Neubauer hemocytometer.

### Fatty acid analysis

Algal cells were harvested by centrifugation at 3500g for 15 min. Fatty acids were extracted and methylated as described [11] with minor modifications. A 15 mL aliquot of algal culture was harvested; following methylation the heptane fraction was concentrated and re-suspended in 40 μL solvent prior to injection of 1 μL on to the GC column. Methyl ester derivatives of total fatty acids extracted were analysed by GC (Agilent 7890A) using an Agilent DB-225 column and identified using known standards (Sigma 37 FAMEs mix).

### Lipid analysis

Total lipid was extracted using a modified Bligh and Dyer method [12]. Freeze dried cells (10 mg) from the stationary growth phase were heated in 1 mL propan-2-ol at 80°C for 10 minutes. PBR samples were extracted from day 42 of culture and RP samples from days three and five. The solvent fraction was retained and the cell pellet homogenised in a total volume of 6 mL chloroform:methanol (2:1). Following centrifugation the cell pellet was re-extracted with 4 mL chloroform:methanol (2:1). Lipid extract was dried under nitrogen and re-susupended in 0.5 mL chloroform. Lipid extract was fractionated into neutral lipids, glycolipids and phospholipids using Sep-Pak columns (Waters Associates, Milford, Massachusetts) as described previously [13]. Fatty acids were methylated and analysed by GC-FID. 17:0 fatty acid was used as an internal standard.

## Results

### Assessment of omega-3 LC-PUFAs accumulation in Pt_Elo5 transgenic cultures grown in 3.5 L bubble columns under varying culture conditions

Commercial production of omega-3 from Pt_Elo5, or any other microalgae, is unlikely to involve media that are expensive and/or difficult to make, therefore the effect of different media, carbon sources and illumination on omega-3 LC-PUFAs production by WT and transgenic Pt_Elo5 strain grown in 3.5 L bubble columns was investigated. Utilising ESAW basal salts and nutrients (ES+EN), we obtained a baseline EPA/DPA/DHA content for Pt_Elo5 of 8.6%/2.9%/5.4% of total fatty acids (16.9% of total fatty acids as omega-3 LC-PUFAs) (S1 Table). The change from ESAW basal salts to Instant Ocean (IO) media improved production levels of EPA/DPA/DHA to 9.8%/3.3%/6.0% (19.0% of total fatty acids as omega-3 LC-PUFAs). The highest content of omega-3 LC-PUFAs was observed when IO and ESAW basal salts were supplemented with F/2 nutrients, in IO+F/2N and ES+F/2N media (21.5% and 21.1%, respectively, of total fatty acids as omega-3 LC-PUFAs). However, the highest levels of DHA accumulated in transgenic strain grown in IO+F2N medium (7.5% of total fatty acids). In WT cells the highest level of EPA was observed in ES+F/2N medium (19.6% of total fatty acids) and only minor presence of DHA (1.5%) has been detected.

**Fig 1:**
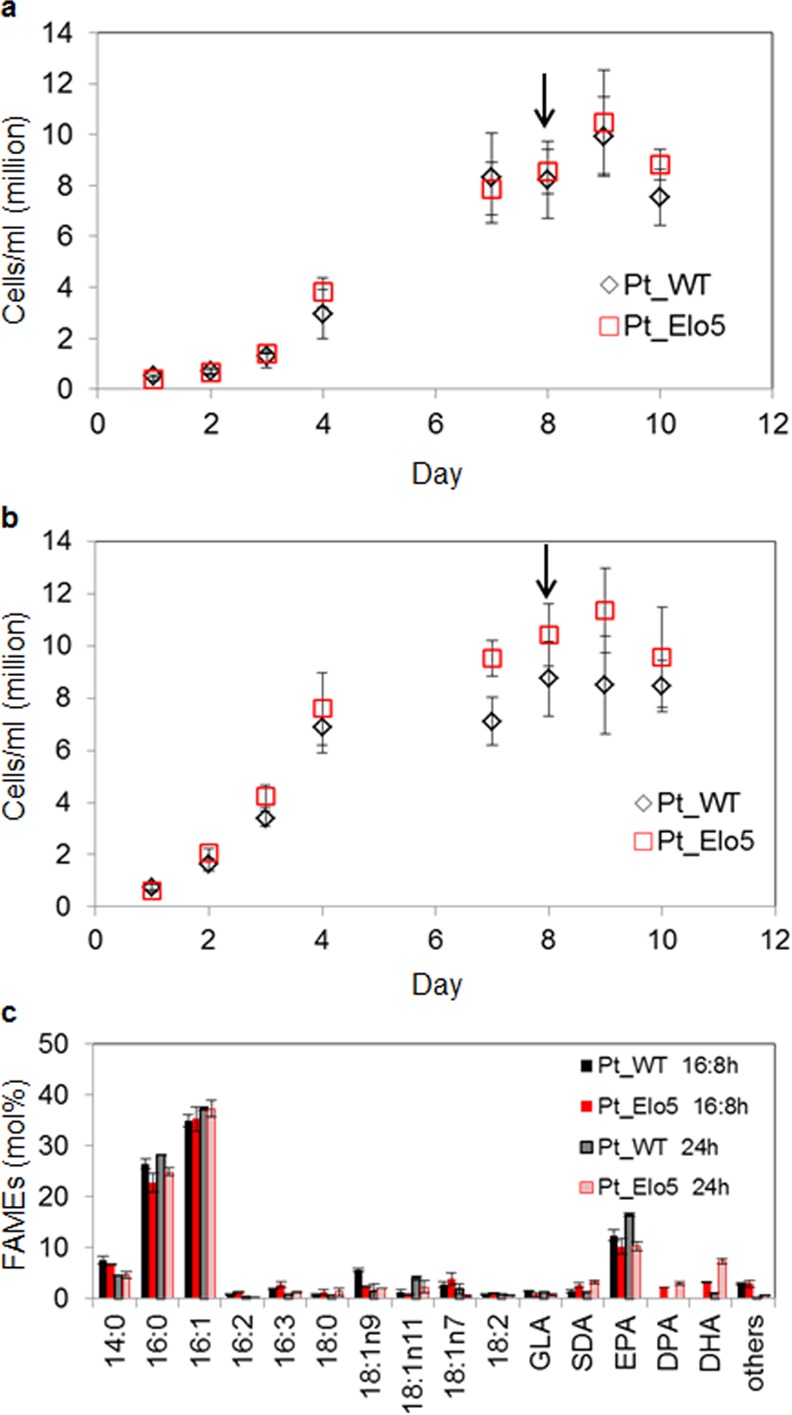
The effect of light intensity on omega-3 LC-PUFAs accumulation_in Pt_Elo5 transgenic cultures grown in 3.5 L bubble columns. Cellular growth of WT (Pt_WT) and transgenic strain Pt_Elo5 cultivated under 16:8 light: dark cycle (a) and continuous light (b). Fatty acid composition of WT and transgenic Pt_Elo5 strains grown under 16:8 light: dark cycle and continuous light (c). Values are the average of three experiments (± standard error). Arrows indicate days in culture when cells were harvested.

Following the identification of IO+F/2N as a promising medium composition, we investigated the effects of light on omega-3 LC PUFAs production of both WT and transgenic strains in 3.5 L bubble columns. WT and transgenic strains were cultivated under continuous light or under light:dark cycle conditions (16:8 h) and cells were sampled in the stationary phase. Exposure to continuous light resulted in enhanced accumulation of omega-3 LC-PUFA (Fig 1). In transgenic cells grown under continuous light, levels of DHA averaged 7.5% of total fatty acids (compared to 2.7% in cells grown under 16:8 h conditions), levels of EPA averaged 10.3% (9.0% under 16:8 h conditions) and DPA comprised 3.7% (2.2% under 16:8 h conditions). In WT cells grown under continuous light conditions the levels of EPA constituted up to 15.5%, compared to 11.4% under 16:8 h conditions. Similar results were obtained when cells were grown in IO+EN media (S2 Table).

**Fig 2:**
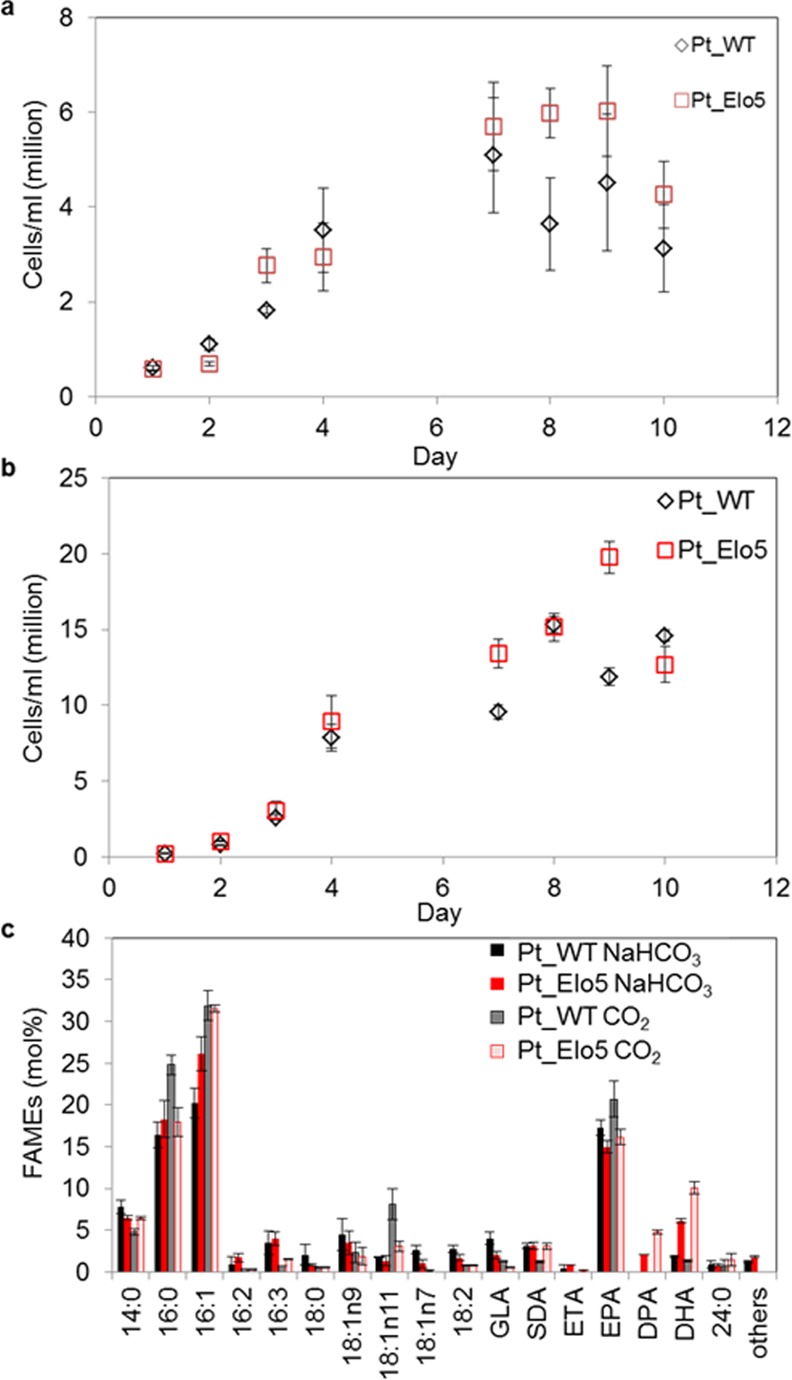
Effect of bicarbonate supplementation and CO_2_ bubbling on omega-3 LC-PUFAs production. Cells were grown in IO+F/2 N under constant light in 3.5 L bubble column and analysed at stationary phase. Cellular growth of WT (Pt_WT) and transgenic strain Pt_Elo5 cultivated in the presence of bicarbonate (a) or CO_2_. Fatty acid composition of WT and transgenic Pt_Elo5 strains grown in the presence of NaHCO_3_ or CO_2_ (c). Values are the average of three experiments (± standard error). Arrows indicate day in culture when cells were harvested.

Addition of bicarbonate salts and CO_2_ bubbling have been shown to positively affect accumulation of lipids in *P*. *tricornutum* cultures [14–16]. In this study the bicarbonate effect was compared to the CO_2_ bubbling and was assessed at stationary (S) phase of the cultures grown in 3.5 L bubble columns in the presence of 15 mM NaHCO_3_ or air supplemented with 1% CO_2._ Both sodium bicarbonate and CO_2_ were added to the cultures from day 1. In agreement with previous results [16] adding bicarbonate slowed cell growth of WT and Pt_Elo5 (Fig 2, S3 Table). Supplementation of culture medium with 15 mM NaHCO_3_ did not alter the levels of omega-3 LC-PUFAs compared to unsupplemented media (Fig 1C). However, increased biomass production and content of EPA, DPA and DHA in transgenic strain Pt_Elo5 and WT cells was observed when the air was supplemented with 1% CO_2_, in agreement with previous studies [14]. Maximum DHA levels averaged 10% (compared to 7.3% in the cultures supplemented with bicarbonate), EPA levels increased to an average of 16.1% (versus 10.1% in cultures supplemented with bicarbonate). The average production of DHA corresponded to 5.6 μg/mg dry weight.

### Omega-3 LC-PUFAs production in Pt_Elo5 transgenic cultures grown in PBR and Raceway Pond

**Fig 3:**
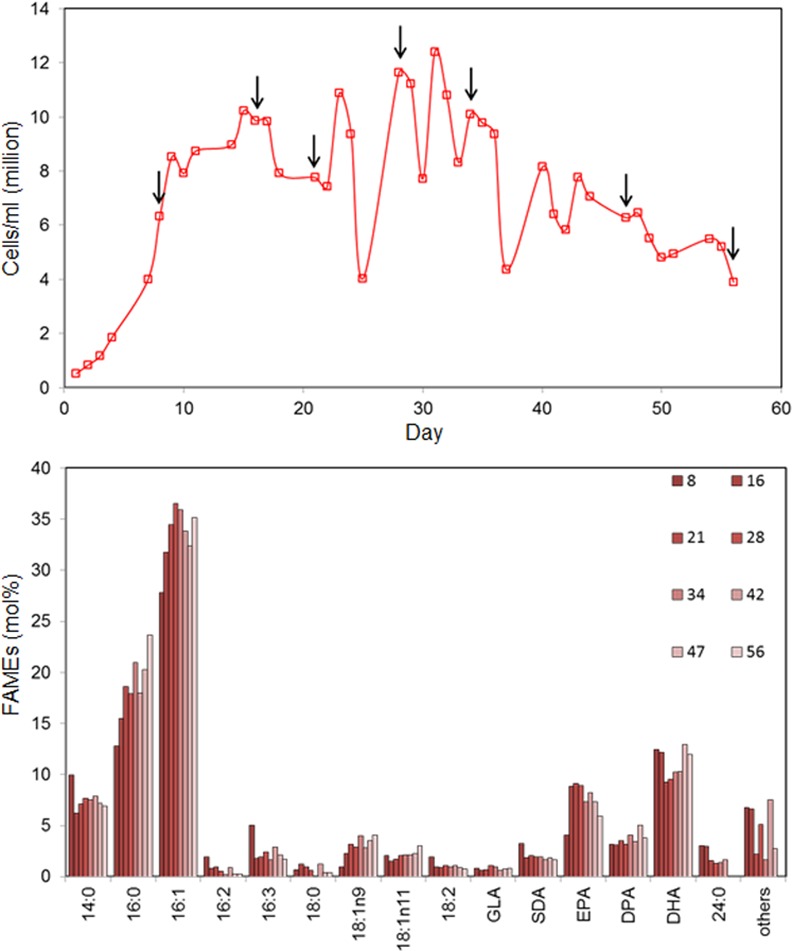
Fatty acid composition of transgenic Pt_Elo5 cells grown in PBR at 16:8 h conditions in IO+F/2 medium. a) Cell concentration, b) Fatty acid composition. Arrows indicate days in culture when cells were harvested.

Following the smaller-scale trials in 3.5 L bubble columns we compared the biomass productivity and omega-3 LC-PUFAs content of the transgenic strain Pt_Elo5 grown in a larger fence-style tubular photobioreactor and a raceway pond. Transgenic cultures were grown in a 550 litre working volume photobioreactor which offers control over pH, lighting, improved agitation and CO_2_ delivery. Cultures were monitored for nearly 2 months (Fig 3). Cellular growth was similar to that observed in 3.5 L bubble column. For the first time we observed a higher DHA production over EPA, with DHA content ranging from 9.5 to 13.0% and EPA levels ranging from 4.0 to 9.1%, respectively, of total fatty acids. Levels of DPA in these experiments averaged 3.7%. The average production of DHA corresponded to 6.4 μg/mg dry weight in a mid-exponentially growing algal culture.

**Fig 4:**
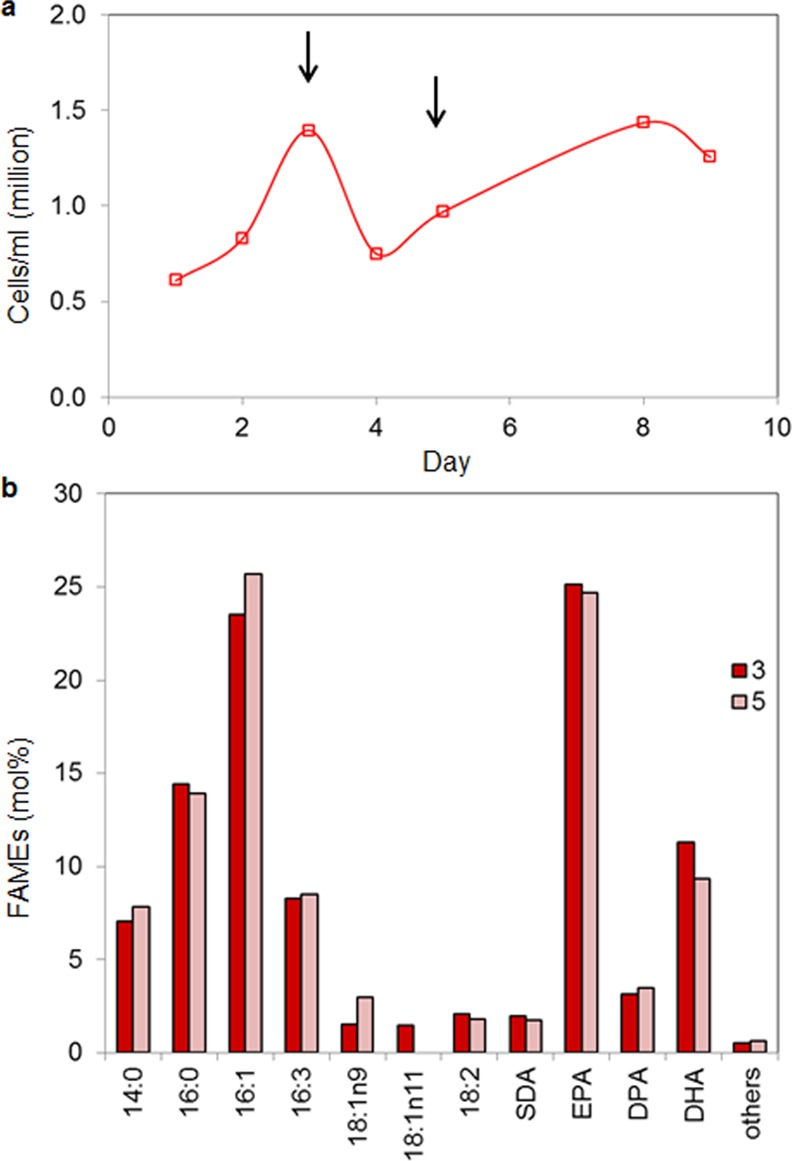
Fatty acid composition of transgenic Pt_Elo5 cells grown in Raceway Pond at 16:8 h conditions in NSW+F2 medium. a) Cell concentration, b) Fatty acid composition. Arrows indicate day of cultivation when cells were harvested.

Cell growth and the EPA:DHA ratio in Pt_Elo5 cells grown in a raceway pond were different from that of the photobioreactor. We observed reduced growth rate and lower cell density of transgenic cells growing in a raceway pond. However, during the whole period of observation, the levels of EPA were significantly higher than DHA, ranging from 24.6% to 25.1% and 9.3% to 11.3%, respectively (of total fatty acids) (Fig 4). This difference in EPA:DHA is unlikely to be due to the substitution of artificial for natural seawater as previous experiments in 3.5 L bubble columns comparing these media types did not impact FA levels in this way (data not shown). DPA content averaged 3%, similar to that observed in the photobioreactor. Levels of 16:0 and 16:1 fatty acids were decreased to an average of 13.9% and 24.2% compared to 20.4% and 34.7% in the photobioreactor, respectively. The average production of DHA corresponded to 2.6 μg/mg.

### Lipid analysis

To further characterize the accumulation and partitioning of omega-3 LC-PUFAs in transgenic strain grown at different conditions, a more detailed lipid analysis was performed on the cultures grown in 100 mL flasks, 3.5 L bubble columns, photobioreactor and raceway pond. Cells in 100 mL flasks and 3.5 L columns were in the S phase. The PBR sample was taken from day 42 of the culture and RP samples from days three and five. In 100 mL flask cultures of wild type *P*. *tricornutum*, EPA was a major constituent of glycolipids (GL) and phospholipids (PL) representing 34.9% and 24.8% respectively and accounted for 15.7% of neutral lipids (NL) (Table 1). DHA represented 8.3% of PL and less than 1% (0.5%) of both GL and NL classes. Lipid analysis of transgenic cultures indicated that the highest levels (28.8%) of DHA accumulated in PL, compared with 5.4% in NL and 2.6% in GL. Conversely, the accumulation of EPA was significantly decreased in PL and NL (6.9% and 4.9% respectively). The levels of EPA in GL fractions of transgenic strain (27.7%) demonstrated a minor reduction compared with those in WT.

**Table 1:** Fatty acid composition of total lipids, neutral lipids and polar lipids isolated from Pt_WT and Pt_Elo5 grown in different cultivation systems (mol %).

	14:0	16:0	16:1	18:0	18:1	EPA	DPA	DHA	24:0	Others
100ml										
**Pt_WT**										
Cells	5.6±0.4	17.3±0.5	37.3±1.7	0.4±0.1	6.2±0.4	21.8±0.6	nd	1.4±0.2	1.7±0.6	6.5±0.3
PL	8.0±0.7	14.2±0.6	20.3±0.9	1.1±0.4	12.7±2.8	24.8±0.7	nd	8.3±1.1	3.1±0.1	0.4±0.2
GL	1.5±0.1	14.8±0.1	27.7±0.6	8.5±0.8	5.2±0.8	34.9±0.7	nd	0.5±0.1	0.2±0.2	0.7±0.2
NL	3.7±0.1	18.6±0.3	46.1±0.8	3.4±0.9	7.6±0.4	15.7±0.6	nd	0.5±0.1	0.5±0.1	0.4±0.1
**Pt_Elo5**										
Cells	6.4±0.6	17.1±0.8	37.2±2.4	0.3±0.1	5.4±0.5	11.1±1.1	1.7±0.1	8.0±0.7	1.3±0.3	6.7±0.3
PL	7.2±1.6	16.4±1.7	18.2±2.0	2.3±1.6	4.5±0.3	6.9±0.4	4.6±0.8	28.8±3.6	3.1±0.5	0.9±0.1
GL	1.6±0.4	13.8±2.7	29.0±2.2	9.2±1.3	4.7±1.2	27.7±1.6	0.5±0.1	2.6±0.1	0.3±0.1	8.0±0.8
NL	3.4±0.3	17.9±0.6	45.4±0.3	3.2±0.3	8.34±1.9	4.9±0.2	2.0±0.1	5.4±0.5	nd	4.2±0.1
**CO**_**2**_										
**Pt_WT**										
Cells	4.7±0.4	24.7±1.2	31.8±1.8	0.5±0.1	10.4±1.9	20.7±2.2	1.3±0.1	0.7±0.7	0.1±0.1	2.1±0.1
PL	7.3±0.7	18.6±1.0	16.5±0.5	1.2±0.1	14.7±0.5	25.8±1.9	nd	2.9±1.4	4.2±1.0	5.2±0.1
GL	2.4±0.1	24.2±0.2	27.0±0.8	11.5±0.1	3.0±0.2	21.6±0.3	nd	0.3±0.1	0.2±0.2	6.7±0.5
NL	4.4±0.1	29.9±0.1	38.0±0.1	2.6±0.3	2.4±0.1	11.7±0.1	nd	0.4±0.1	nd	1.9±0.1
**Pt_Elo5**										
**Cells**	6.4±0.2	17.9±1.7	31.3±0.4	0.5±0.1	5.21±0.1	16.1±0.9	4.7±0.7	10.0±0.7	1.5±0.1	2.3±0.1
PL	9.9±0.5	19.2±0.1	19.5±0.2	0.9±0.1	4.1±0.1	8.4±0.5	8.4±0.8	18.7±0.8	5.2±0.2	2.0±0.1
GL	2.3±0.1	21.0±0.6	30.0±0.6	9.8±0.4	2.6±0.1	24.0±1.0	0.7±0.4	1.8±0.2	0.4±0.1	5.0±0.2
NL	4.8±0.4	28.5±1.0	39.0±1.0	3.1±0.3	1.9±0.6	7.8±0.9	2.3±0.1	3.6±0.1	nd	2.0±0.2
**PBR**										
Cells*	6.6±0.6	19.8±0.3	34.1±1.1	0.37±0.1	6.3±0.3	8.7±0.5	4.5±0.3	12.3±0.7	0.4±0.1	5.3±0.2
PL	14.7±0.4	20.3±0.4	20.5±0.3	0.4±0.2	4.4±0.1	6.0±0.5	4.5±0.5	16.4±0.6	1.1±0.5	3.6±0.1
GL	4.6±0.4	16.1±0.2	34.8±0.1	4.7±0.5	3.8±0.2	21.8±0.5	0.9±0.1	2.9±0.1	0.7±0.5	2.1±0.1
NL	6.8±0.1	18.9±0.8	31.2±0.5	7.7±1.1	5.5±0.1	6.7±0.2	3.4±0.1	7.1±0.1	1.3±0.1	3.0±0.1
**RP**										
Cells**	7.4±0.3	14.1±0.3	24.5±1.1	nd	3.4±0.7	24.8±0.3	3.3±0.2	10.3±1.0	nd	10.4±0.1
PL	9.4±0.7	15.5±2.1	25.0±1.5	2.1±0.3	5.7±0.7	10.3±1.9	6.1±0.3	16.0±2.0	1.8±0.2	6.5±0.3
GL	2.4±0.1	22.5±2.3	16.0±0.6	16.5±2.5	2.2±0.4	19.9±3.4	0.6±0.1	2.3±0.2	0.3±0.1	13.6±0.4
NL	3.9±0.2	21.9±0.25	13.0±0.2	31.1±0.7	4.1±0.7	12.8±1.0	0.4±0.1	0.6±0.1	nd	10.7±0.8

^+^ *Other lipids* Lipids comprising <2% of the total algal lipids. GL, glycolipid; NL, neutral lipid; PL, phospholipid.

* Average of PBR samples from day 25, 35 and 43.

**Average of RP samples from day 3 and 5.

The pattern of omega-3 LC-PUFAs distribution in the cells of cultures grown in 3.5 L bubble columns in the presence of CO_2_ was similar to those grown in 100 mL flasks. Analyses of neutral and polar lipids of transgenic strain revealed that EPA was predominantly accumulated in the glycolipid fraction of transgenic strain (averaged 24.0%) and equally distributed between neutral lipids (7.8%) and phospholipids (8.4%), whereas in WT strain EPA comprised 21.6% of total fatty acids in glycolipids, and 25.8% of TFA in phospholipids, averaging 11.7% in neutral lipids. DHA and DPA mainly accumulated in phospholipids of transgenic strain (18.7% and 8.4% respectively) although neutral lipids showed accumulation of DHA and DPA up to 3.6% and 2.3% respectively. Contrary, very low levels of DHA were observed in phospholipids of WT (2.9%). Among other changes in fatty acids distribution in lipid fractions of WT and transgenic cells grown in the presence of CO_2_ the most substantial was a decrease of proportions of oleic acid (OA, C18:1Δ9) in NL and increase of C16:0 in GL an NL.

Analysis of transgenic cells grown in PBR revealed a similar pattern of partitioning of omega-3 LC-PUFAs compared with our observations in 3.5 L bubble columns. EPA was predominantly accumulated in glycolipids (21.8%); DHA levels were the highest in phospholipid fractions (16.4%) followed by neutral lipids (7.1%) and glycolipids (2.9%). EPA levels were equally distributed between neutral lipids (6.7%) and phospholipids (6%).

Lipid composition of cells grown in the raceway pond was similar to those in the photobioreactor, although a higher content of EPA was observed in NL (12.8%). Partitioning of DHA into NL was particularly low (0.6%).

## Discussion

The marine diatom *P*. *tricornutum* has been intensively investigated for the industrial production potential of EPA [3, 14, 17–21]. The majority of studies have been performed at the laboratory scale on the cultures growing in up to 1 L flasks or small bioreactors and open pond systems. Recently, we have engineered *P*. *tricornutum* to accumulate elevated levels of DHA and DPA by overexpressing heterologous genes encoding enzyme activities of the LC-PUFA biosynthetic pathway [10]. In this present study we have explored the enhanced omega-3 production capacity of transgenic strain Pt_Elo5 utilizing four systems: 100 mL cultures, 3.5 L bubble column photobioreactors, a pilot-scale 550 L photobioreactor and an open-pond style raceway system (working volume 1250 L). Light intensity and carbon sources are considered to be major factors affecting cell growth and the production of omega-3 LC-PUFAs in *P*. *tricornutum* [14–16, 22, 23]. Culturing of *P*. *tricornutum* under continuous light or exposure to UV radiation resulted in significant improvement in cell growth and EPA content [22, 23]. We have demonstrated that transgenic cultures grown under continuous light conditions in 3.5 L BC accumulated higher levels of DHA as compared to that grown in light-dark cycle. In accordance with previous reports [22, 23] the supply of light clearly influenced the growth rate and also transgene-derived omega-3 production–in this latter case, this is most likely due to the light-responsive fucoxanthin chlorophyll a/c binding protein (*fcp*) promoter used to drive the expression of the recombinant elongase gene. However, it is unlikely that continuous light is economically viable at the industrial scale, even though continuous illumination could potentially increase DHA production further, and with regards to the health benefits of the derived omega-3 product further optimisation may seek specific DHA: EPA ratio. Alternative strategies to improve illumination efficiency and decrease the cost of microalgal production may involve mixotrophic or heterotrophic growth in the presence of suitable organic nutrients.

The highest EPA productivity of 43.13 mg L^−1^ per day was obtained in the flask cultures of 1 L working volume grown under low irradiance (165 μE m^−2^s^−1^) in the presence of 0.1 M glycerol and supplemented periodically with urea 0.01 M [24]. This yield was 13-fold higher than the maximum EPA productivity obtained in the photoautotrophically grown control [17] and reported similar yields (47.8 mg L^−1^ per day) for photoautotrophic growth on glycerol of this same strain in 50–200 L working volume outdoor pilot-scale tubular photobioreactors (tube diameter 30 and 60 mm respectively), which suggest the possibility of using mixotrophy for the mass production of microalgae.

More relevantly, Mus et al. [16] recently demonstrated that the addition of 15 mM bicarbonate to *P*. *tricornutum* cultures had a positive effect on lipid accumulation. We compared the effect of culture gassing with 1% (vol/vol) carbon dioxide and supplementation of culture medium with 15 mM NaHCO_3_ on the content of omega-3 LC-PUFAs. Improved cell growth and the increased omega-3 LC- PUFAs content of WT and transgenic cells (30.8%) was observed when the air was supplemented with 1% CO_2_ (Fig 2), confirming the benefits of this addition.

**Fig 5:**
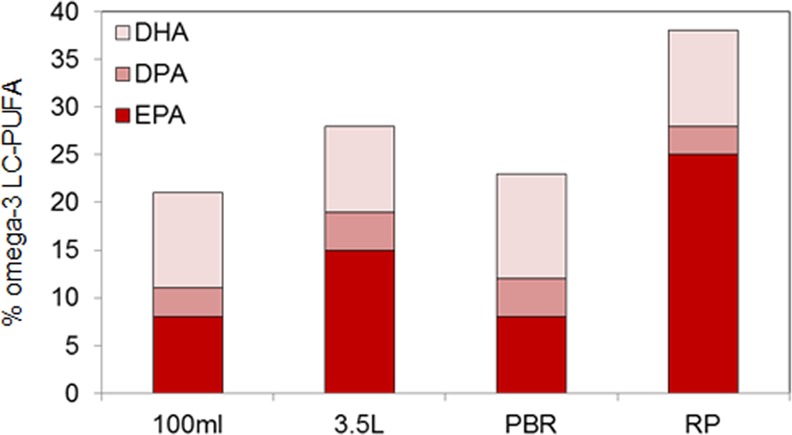
Comparison of omega-3 LC-PUFAs content in different production systems.

In this study we assessed omega-3 LC-PUFAs production in Pt_Elo5 transgenic cultures grown in photobioreactors and raceway ponds. We have shown that transgenic strain Pt_Elo5 was successfully cultivated at large scale, demonstrating the efficacy of producing omega-3 LC-PUFAs in bioreactors and raceway ponds. The DHA content of cultures grown in PBR ranged from 10.2 to 13.6%, EPA levels ranged from 8 to 11% and DPA averaged 5%. For the first time, we observed a higher DHA production over EPA, generating an average EPA/DPA/DHA profile of 8.7%/4.5%/12.3%, with an omega-3 content up to 25.5% (Fig 5). The DHA production corresponded to 6.4 μg/mg dry weight in a mid-exponentially growing algal culture. Whilst the media and nutrients were identical to those of the smaller scale trials, the improved agitation and light exposure (despite a 16:8 h cycle), and subsequent omega-3 production, within such a system offers a clear opportunity for the development of Pt_Elo5 as a platform for commercial production. Whilst initial trials in the 3.5 L bubble column systems (PAR intensity of 80 μmol photons m^-2^s^-1^) show that modifications to the light cycle can be used to optimise DHA production, results from the tubular bioreactor (PAR intensity of 450 μmol photons m^-2^s^-1^) suggest that light intensity is perhaps more important. This observation most likely has its roots in the use of the *fcp* promoter to drive the expression of the transgene [10].

Despite the clear potential for production within closed photobioreactor systems, the majority of commercial algal biomass is still produced within open pond systems. Culturing of microalgae in open ponds has been well established but only a few species can be maintained in conventional open systems. Benavides et al [25] compared the biomass productivity and fatty acid composition of *P*. *tricornutum* grown outdoors in open ponds and photobioreactors (PBRs). The productivity of cultures was higher in photobioreactors compared to that in open ponds, most likely as a result of a better light-dark regime experienced by the cells in short light-path tubular PBRs, which may have allowed a more efficient use of light. The fatty acid profile of the biomass did not change significantly between the biomass grown in the two systems though some minor differences were found in the amount of DHA (average 2.4% in PBR and 1.1% in RP).

We trialled the GM strain in an internally housed 1250 litre raceway pond mimic (thereby offering closed containment). Omega-3 production, at ambient temperature but supplemented with artificial illumination to a PAR of 110 μmol photons m^-2^s^-1^ on a 16:8 h cycle, in natural seawater and F/2 nutrients was an impressive 24.8%/3.3%/10.3% (EPA/DPA/DHA). In RP cultures EPA yield was notably enhanced averaging 25% and the total content of omega-3 LC-PUFAs (38.4%) was higher than that in PBR (25.5%), thereby demonstrating the potential for Pt_Elo5 to fit into existing raceway pond based production pipelines. However, the average production of DHA was the lowest in RP and corresponded to 2.6 μg/mg dry weight. Given that RP environments give typically lower yields than closed PBR systems this is not surprising, however optimisation of growth conditions such as inoculating cultures with a higher cell density may reduce bacterial contamination and increase yields. Bacterial contamination can compete for nutrients resulting in inhibited algae growth; severe contamination can lead to culture deterioration through the production of toxic metabolites. The maintenance of monocultures and/or control of bacterial population growth through manipulation of culture conditions is therefore desirable.

Considering the on-going discussion on the bioavailability of different omega-3 sources (phospholipids, triglycerides and ethyl esters), studies on localization of LC-PUFAs in the lipid pool and, especially, partitioning into TAGs represent critical aspects for the commercialization. Most commonly, LC-PUFA in microalgae are esterified into membrane phospholipids [26], while it is generally accepted that esterification into TAGs would provide a more suitable dietary source for the human diet and simplify the purification of these fatty acids from microalgal biomass. Culture conditions including medium composition, light intensity, aeration, culture age and growth systems (indoors, outdoors, photobioreactors and raceway ponds) have significant effects on fatty acids content and partitioning [17, 19, 20, 27–30]. Thus, under optimal conditions tested in a continuous flow reactor, 84% of EPA was found in galactolipids (monogalactosyldiacylglycerols) and 11% EPA in TAGs [19]. Similar results were reported by Arao et al [31] who had found only minor percentage of EPA in TAGs. Alonso et al [20] studied the influence of culture age and nitrogen concentration on the distribution of fatty acids among the different acyl lipid classes, reporting 44.7% of EPA in TAGs in cultures grown indoors. Ryckebosch et al [30] demonstrated that EPA was more abundant in the neutral lipids (31.7%) of *P*. *tricornutum* cells grown in 130 L bioreactor under continuous light (125 μmol photons m^-2^ s^-1^). Differences in preferential deposition of this fatty acid either in neutral or in polar lipids reflect the importance of cultivation conditions. The vast variation of data on fatty acids distribution among different acyl lipid classes of *P*. *tricornutum* clearly demonstrated the importance of performing detailed analysis of fatty acid production and partitioning under controlled growth conditions.

We analysed fatty acids distribution in the Pt_Elo5 transgenic cultures grown in 100 mL, 3.5 L BC, 550 L PBR and 1250 L RP. EPA content of Pt_ELo5 cells cultivated in different systems was highest in glycolipids, but evenly distributed between phospholipids and neutral lipids. We found that Pt_Elo5 grown in RP produced the highest levels of EPA (12.8%) incorporated in neutral lipids (Table 1). In accordance with previous results the levels of DHA were the highest in the phospholipids (reaching maximum 28.8% in transgenic cells, growing in 100 mL culture) followed by neutral lipids and glycolipids. The highest partitioning of DHA in neutral lipids was observed in cultures grown in PBR (7.1%) and just 0.6% constituted to the neutral lipids of cultures grown in RP. Tonon et al. [29] found that *P*. *tricornutum* grown in batch culture produced DHA but did not incorporate this LC-PUFA into TAGs, which is in contrast to the report of Alonso et al. [20] who found a low quantity of DHA in the storage lipids under continuous culture conditions. Therefore, culture growing conditions have significant impact on DHA content and distribution in lipid classes, an important consideration for any industrial scale production process.

There is little precedent for the large scale cultivation of genetically modified microalgae. This may change with the increased interest in the exploitation of genetically modified microalgae, which will presumably require a more suitable containment infrastructure (and associated legislation) to that offered by growth in contained photobioreactors. However, recent small scale trials by Sapphire Energy at the 600–800 litre scale with GM microalgae in open mini-ponds suggest such activities may gain public and governmental acceptance in the near future. Originally the expression of additional elongase function in *Phaeodactylum* was targeted at simply improving DHA production capacity to an ‘acceptable’ amount in a strain established in the aquaculture industry. However, these results clearly show the potential for achieving desired EPA: DHA ratios and move Pt_Elo5 beyond a simple proof-of-principle system into the realms of commercial relevance. Further strain improvement process will likely involve a combination of metabolic engineering to enhance the levels of omega-3 LC-PUFAs accumulation in TAG, optimization of growth and oil production, and downstream processing to reduce the costs of oil extraction. Trophic conversion may represent an alternative approach in the area of upstream processing to improve light efficiency and cultivation mode for commercial exploitation.

The advent of the synthetic biology era will, without doubt, change the face of industrial biotechnology, as more genetically modified systems become available for exploitation. Public and governmental acceptance of large scale culturing is not a given, in particular the environmental implications of open pond culture. Regardless of the route taken (open or closed culture), Pt_Elo5 appears well suited to become a versatile industrial platform for the production of bespoke omega-3 formulations.

## Supporting Information

S1 Table The effect of media composition on omega-3 LC-PUFAs production. Baseline data for EPA, DPA and DHA levels in WT and transgenic diatoms grown in different media (mol %).
(DOCX)

S2 Table Effect of light conditions on EPA, DPA and DHA accumulation. The effects of different light conditions on accumulation of EPA, DPA and DHA in WT and transgenic diatoms (mol %). Cells were exposed to a 16:8 h cycle or constant light.
(DOCX)

S3 Table The effect of bicarbonate supplementation and CO_2_ bubbling on omega-3 LC-PUFAs accumulation. The effects of different carbonic supplementation on accumulation of EPA, DPA and DHA in WT and transgenic diatoms (mol%)
(DOCX)
