# Supplementary material for: Towards the Industrial Production of Omega-3 Long Chain Polyunsaturated Fatty Acids from a Genetically Modified Diatom Phaeodactylum tricornutum
Source: PLoS One. 2015 Dec 14;10(12):e0144054. doi: 10.1371/journal.pone.0144054 (PMC4681182; doi:10.1371/journal.pone.0144054)
Supplement: S1 Table — Baseline data for EPA, DPA and DHA levels in WT and transgenic diatoms grown in different media (mol %). (DOCX) [file pone.0144054.s001.docx]

| Cell type | Media | EPA | DPA | DHA |
| --- | --- | --- | --- | --- |
|  |  |  |  |  |
| WT  Elo5 | IO+EN  IO+F/2N  ES+EN  ES+F/2N  IO+EN  IO+F/2N  ES+EN  ES+F/2N | 14.4±0.8  15.5±1.6  12.7±1.0  19.6±1.7  9.8±0.9  10.3±0.1  8.6±0.2  10.9±0.8 | nd  nd  nd  nd  3.3±0.2  3.7±0.5  2.9±0.2  4.6±0.2 | 1.2±0.1  1.3±0.2  1.4±0.2  1.5±0.2  6.0±0.1  7.5±0.4  5.4±0.2  5.6±0.3 |
