# Supplementary material for: Towards the Industrial Production of Omega-3 Long Chain Polyunsaturated Fatty Acids from a Genetically Modified Diatom Phaeodactylum tricornutum
Source: PLoS One. 2015 Dec 14;10(12):e0144054. doi: 10.1371/journal.pone.0144054 (PMC4681182; doi:10.1371/journal.pone.0144054)
Supplement: S2 Table — The effects of different light conditions on accumulation of EPA, DPA and DHA in WT and transgenic diatoms (mol %). Cells were exposed to a 16:8 h cycle or constant light. (DOCX) [file pone.0144054.s002.docx]

| Cell type | Media | Light conditions | EPA | DPA | DHA |
| --- | --- | --- | --- | --- | --- |
| WT  Elo5 | IO+EN  IO+F/2N  IO+EN  IO+F/2N | 24  16:8  24  16:8  24  16:8  24  16:8 | 14.4±0.8  10.9±0.2  15.5±1.6  11.4±0.1  9.8±0.9  6.3±0.2  10.3±0.1  9.0±0.1 | nd  nd  nd  nd  3.3±0.2  1.4±0.1  3.7±0.5  2.0±0.1 | 1.2±0.1  0.5±0.1  1.3±0.2  nd  6.0±0.1  2.1±0.1  7.5±0.4  2.7±0.1 |

*.*
