# Supplementary material for: Towards the Industrial Production of Omega-3 Long Chain Polyunsaturated Fatty Acids from a Genetically Modified Diatom Phaeodactylum tricornutum
Source: PLoS One. 2015 Dec 14;10(12):e0144054. doi: 10.1371/journal.pone.0144054 (PMC4681182; doi:10.1371/journal.pone.0144054)
Supplement: S3 Table — The effects of different carbonic supplementation on accumulation of EPA, DPA and DHA in WT and transgenic diatoms (mol%) (DOCX) [file pone.0144054.s003.docx]

| Cell type | Addition | EPA | DPA | DHA |
| --- | --- | --- | --- | --- |
| WT  Elo5 | No addition  Bicarbonate  CO_2_  No addition  Bicarbonate  CO_2_ | 15.5±1.6  16.3±0.3  22.0±0.8  10.3±0.1  10.1±0.8  16.0±0.9 | nd  nd  nd  3.7±0.5  2.9±0.3  4.7±0.3 | 1.3±0.2  1.1±0.1  1.3±0.2  7.5±0.4  7.3±0.6  10.0±0.7 |
